# Smoke-Free Laws and Direct Democracy Initiatives on Smoking Bans in Germany: A Systematic Review and Quantitative Assessment

**DOI:** 10.3390/ijerph110100685

**Published:** 2014-01-03

**Authors:** Stefan Kohler, Philipp Minkner

**Affiliations:** Institute for Social Medicine, Epidemiology and Health Economics, Charité University Medical Center, Berlin 10117, Germany; E-Mail: philipp.minkner@charite.de

**Keywords:** anti-smoking law, court decision, Germany, petition, popular initiative, referendum, smoking ban, smoke-free law, smoking prevalence, tobacco control

## Abstract

*Background:* Germany’s 16 states regulate smoking differently within health protection principles laid down in the federal law. All state smoke-free laws in Germany have undergone at least one change since taking effect. *Methods:* We systematically review federal and state laws regulating smoking, as well as petitions, popular initiatives and referenda that aimed at changing statutory smoking bans. Data generated through the systematic review were correlated with state smoking rates. *Results:* The protection from the dangers of secondhand smoke is the primary motive for smoking bans in Germany. The first smoke-free laws affecting smoking in pubs, restaurants and several other public places were introduced in 2007. In 2008, the Federal Constitutional Court of Germany ruled in a leading decision on the smoke-free laws of two states that some common smoking ban exemptions of the introduced smoke-free laws violate the basic right to freely exercise a profession and mandated revisions. All states but Bavaria and Saarland, whose smoking bans were more and less comprehensive than those judged by the constitutional court, respectively, needed to change the smoking ban exemptions to reconcile their smoke-free laws with the constitution. Direct democracy initiatives to change smoking bans were only successful in Bavaria in 2010, but a total of 15 initiatives by citizens’ or interest groups attempted to influence non-smokers protection legislation through direct democratic procedures. Early ratification of a smoking ban in a federal state correlates with a higher reduction in the smoking rate from 2005 to 2009 (Spearman’s ρ = 0.51, *p* = 0.04). *Conclusions:* The federal government structure and direct democratic participation in smoke-free legislation in Germany has produced a diversity of local smoking bans and exemptions.

## 1. Introduction

More than 3,300 people are estimated to die annually from secondhand tobacco smoke in Germany [1]. According to a 2009 addiction survey, the highest exposure of non-smokers to secondhand smoke is at the workplace and during non-domestic leisure activities [2]. A percent of 47.4 of females and 36 percent of males in Germany feel strongly disturbed by tobacco smoke [3]. Tobacco smoke contains about 4,800 chemicals. Of these, about 200 are toxic and about 50 are classified as carcinogenic [4,5]. The harmful substances contained, such as benzene, formaldehyde and nitrosamines, affect the active consumers of tobacco and people who passively inhale tobacco smoke.

Several countries have banned smoking in public places to protect non-smokers from secondhand tobacco smoke. Germany has ratified the WHO Framework Convention on Tobacco Control (FCTC) on 12 December 2004. The FCTC was developed in response to the globalization of the tobacco epidemic. Its signatories assumed an obligation to reduce the demand for and the supply of tobacco, and its eighth article on the protection from exposure to tobacco smoke explicitly states: “(1) Parties recognize that scientific evidence has unequivocally established that exposure to tobacco smoke causes death, disease and disability; (2) Each Party shall adopt and implement in areas of existing national jurisdiction as determined by national law and actively promote at other jurisdictional levels the adoption and implementation of effective legislative, executive, administrative and/or other measures, providing for protection from exposure to tobacco smoke in indoor workplaces, public transport, indoor public places and, as appropriate, other public places” [6].

The extent and strictness of smoking bans differ within Germany. Each of the 16 federal states of Germany has a different set of regulations for non-smokers protection. The Association of European Cancer Leagues published studies on the effectiveness of political measures against tobacco smoking. Germany ranked 27 out of 30 European countries compared in 2007 and was called the most problematic country in reference to tobacco control [7]. In 2010, Germany ranked 26 out of 31 compared European countries [8]. In this article, firstly, we review the smoke-free laws of Germany and present fundamental court decisions with regard to smoking. Secondly, we compare the regulation of smoking in pubs and restaurants, as the most debated public places in which smoking has become regulated by law, across states. Thirdly, we review the direct democratic activities around smoking bans in Germany and discuss how citizens and interest groups influenced the legislative process on non-smokers protection.

## 2. Materials and Methods

Federal law in Germany is only effective if announced in the *Federal Law Gazette* (Bundesgesetzblatt). The federal states of Germany announce their laws and ordinances in a *Law and Ordinance Gazette* (Gesetz- und Verordnungsblatt). In the state of Saarland an *Official Gazette* (Amtsblatt) fulfills this function. We searched the respective gazettes of the federal republic and all 16 federal states for announcements of smoke-free laws through the *Beck-Online* law database [9]. Officially announced changes of the smoke-free laws were considered at least until 28 June 2013 and at most until 27 November 2013 (supplementary Table S1). We reviewed the full texts of the federal non-smokers protection laws [10,11] as well as the first and most recent versions of the state smoke-free laws [12,13,14,15,16,17,18,19,20,21,22,23,24,25,26,27,28]. Full texts of the smoke-free laws and amendments were accessed through an open access archive for the federal and state law gazettes, called *Parlamentsspiegel* [29]. Smoke-free laws and further information about tobacco control are also available through the website of the *Aktionsbündnis Nichtrauchen e. V.* (Action Alliance for Non-smoking), an alliance of major German health NGOs [30]. Additional information was used to assess if private functions in a pub or restaurant can be exempted from a smoking ban [31,32,33,34].

Petitions, popular initiatives and referendums on non-smokers protection were extracted from the referendum reports for the years 2000 to 2012 [35,36]. The referendum report is a publication by *Mehr Demokratie e. V.* (More Democracy), a German NGO for direct democracy that annually reports on direct democratic activities in Germany since 2000. In addition, all state websites were screened for data on petitions, popular initiatives and referendums on non-smokers protection. 

In a correlation analysis, data generated through the systematic review were combined with disaggregated data on smoking rates. Data on the effective date of the first federal non-smokers protection laws in Germany and fines for breaches of these laws were extracted from the reviewed federal state laws and the federal *Administrative Offence Act* (Ordnungswidrigkeitengesetz) [37]. Smoking rates in the federal states of Germany stem from 2005 and 2009 microcensus data. The microcensus is an annual representative household survey of one percent of the German population (340,000 households with approximately 700,000 persons in 2009) that usually asks about smoking habits every four years. Only persons aged 15 years and above were asked five questions about their smoking habits, including the current smoking status. Answering these microcensus questions is voluntary. We compare the 2005 and 2009 smoking rates reported in the microcensus that are age-standardized to the year 1987 German population [38].

## 3. Results and Discussion

### 3.1. Federal Non-smokers Protection

The protection from the dangers of secondhand smoke is the primary motive for smoking bans in Germany. On 9 February 1998, before there were statutory smoking bans in Germany, the *Federal Constitutional Court* (Bundesverfassungsgericht (BVerfG)) did not accept a constitutional complaint in which the complainant felt violated in his fundamental rights to physical integrity and free development of personality by smoking in places open to the public (BVerfG, 1 BvR 2234/97). On 1 September 2007, the *Law for Protection from the Hazards of Passive Smoking* came into force in the Federal Republic of Germany [11]. This law included various changes of and amendments to the existing legislation and an article imposing a ban on smoking in federal facilities and public transport, named the *Law for the Introduction of a Smoking Ban in Federal Facilities and Public Transport* (*Federal Non-smokers Protection Act*) [10].

The *Federal Non-smokers Protection Act* of the *Law for Protection from the Hazards of Passive Smoking* governs two essential aspects: Firstly, it prohibits smoking in federal facilities as well as in the constitutional bodies of the federation, e.g., government agencies, courts, or federal corporations, institutions and foundations. These institutions, however, were allowed to establish designated smoking zones where tobacco may be consumed. Secondly, the law banned smoking from all modes of public transport (planes, trains, buses, trams, taxis, and passenger ships) and in all train station buildings. If space allows, smoking can be permitted in designated and appropriately marked areas of the train stations. The German Railway extended the statutory smoking ban in their house rules from station buildings to the entire station area with the exemption of designated smoking areas [39]. The federal *Law for Protection from the Hazards of Passive Smoking* also changed the *Youth Protection Act* (Jugendschutzgesetz) [40]. Firstly, the age limit for the purchase of tobacco products has been increased from 16 to 18 years. Secondly, children and young people (below the age of 18) were not to be allowed to smoke in public (§ 10 Jugendschutzgesetz). Furthermore, the federal *Workplace Regulations* (Arbeitsstättenverordnung) were modified by the *Law for Protection from the Hazards of Passive Smoking*. The following sentence was added: The employer shall ban smoking in all or specific areas of the workplace to the extent necessary to effectively protect non-smoking employees from the health hazards of tobacco smoke (§ 5 Abs. 1 Satz 2 (§ 5 subsection 1 sentence 2) Arbeitsstättenverordnung). Workplaces open to the public (and therefore pubs and restaurants) remained excluded from this strict federal regulation and only required to ban smoking to the extent compatible with the nature of the business or type of employment (§ 5 Abs. 2 Arbeitsstättenverordnung) [41].

In addition to the *Law for Protection from the Hazards of Passive Smoking*, other regulations prohibit smoking. There were different approaches to protect non-smokers in the gastronomy sector. In 2005, a non-binding agreement between the Federal Ministry of Health and the German Hotel and Restaurant Association was signed which did not lead to the desired improvement in non-smokers protection [42]. In fact, the federal government declared that it lacked the legislative competence in this area and, since 2007, the non-smokers protection in the gastronomy sector has been regulated by state laws.

Smoking is generally permitted in rented apartments (District Court (Landgericht) Cologne, 9 S 188/98; District Court (Landgericht) Paderborn, 1 S 2/00). If the odor caused by smoking cannot be eliminated by a single airing, then damage compensation can be claimed (District Court (Amtsgericht) Rosenheim, 16 C 1946/93). The landlord may ban smoking in the stairwell (District Court (Amtsgericht) Hannover, 70 II 414/99). Odor trouble caused by passive smoking that occurs during short stays in the stairwell or elevator must be accepted (Labor Court (Arbeitsgericht) Munich, 2 Z BR 105/98). Tenants are entitled to reduce their rent if, due to the design of the house, tobacco smoke from another apartment enters their own apartment and, thus, imposes a health risk or unpleasant odor (District Court (Amtsgericht) Stuttgart-Bad Cannstatt, 6 C 1711/97).

**Table 1:** Smoke-free laws, smoking bans and exemptions for pubs and restaurants, and smoking rates in the federal states of Germany.

Federal State	Code	Effective Date	Changes		Expiry Date	Fine (€)	Pubs and Restaurants	Smoking Rate 2009	% Change since 2005
			Last	#					
Baden-Wuerttemberg	BW	01.08.07	03.03.09	1	None	≤40/2,500	Ban ^a,b^	24.4	−5.1
Bavaria	BY	01.01.08	23.07.10	3	None	(5–1,000)	Ban ^c^	25.6	0.0
Berlin	BE	01.01.08	03.06.10	3	None	≤100/1,000	Ban ^a,b,d^	32.1	−2.7
Brandenburg	BB	01.01.08	15.07.10	2	None	5–100/10–1,000	Ban ^a,b^	30.7	−1.6
Bremen	HB	01.01.08	25.06.13	4	31.07.18	≤500/2,500	Ban ^a,b^	32.1	−4.2
Hamburg	HH	01.01.08	19.06.12	3	None	20–200/50–500	Ban ^a,b^	27.5	−10.4
Hesse	HE	01.10.07	27.09.12	3	31.12.20	≤200/2,500	Ban ^a,bc^	26.5	−3.3
M.-W. Pomerania	MV	01.08.07	17.12.09	1	31.07.14	≤500/10,000	Ban ^a,b^	33.9	−5.6
Lower Saxony	NI	01.08.07	10.12.08	1	None	(5–1,000)	Ban ^a,b^	28.0	−5.7
N. Rhine-Westphalia	NW	01.01.08	04.12.12	2	None	≤2,500	Ban ^c^	28.5	−5.6
Rhineland-Palatinate	RP	15.02.08	26.05.09	1	None	≤500/1,000	Ban ^a,b,c^	27.4	−0.4
Saarland	SL	15.02.08	21.06.10	5	31.12.15	≤200/1,000	Ban ^c^	27.2	−3.5
Saxony	SN	01.02.08	01.07.12	4	None	≤5,000	Ban ^a,b,c^	27.3	0.0
Saxony-Anhalt	SA	01.01.08	23.01.13	4	None	(5–1,000)	Ban ^a,b^	32.9	6.5
Schleswig-Holstein	SH	01.01.08	25.04.09	1	None	≤1,000	Ban ^a,b^	29.0	−7.3
Thuringia	TH	01.07.08	31.12.12	2	None	20–200/50–500	Ban ^a,b^	30.3	2.0

Notes: Dates are denoted in the format date month year. Changes denote the announcement date of the last change to the smoke-free law and the total number of changes made since the effective date (see supplementary Table S1). Fine denotes fines established in the smoke-free law that can be imposed for minor breaches of the law by violating/not enforcing ban. Parentheses indicate that the amount of the fine is regulated in the § 17 *Administrative Offence Act* (Ordnungswidrigkeitengesetz). Superscripts denote smoking ban exemptions for pubs and restaurants: ^a^ Separate rooms; ^b^ Smoking pub; ^c^ Smoking can be permitted while hosting a *private function* (geschlossene Gesellschaft); ^d^ Operated and visibly labeled as shisha lounge in which no alcoholic beverages are served. %-Change denotes the change in the age-standardized smoking rate from 2005 to 2009 as a percentage of the baseline year 2005. The HB non-smokers protection act replaced an earlier law from 18.07.06 that partially banned smoking in hospitals, daycare for children, and schools. The last change in BY replaced the non-smokers protection act completely after a referendum. Repeated offenders can face higher fines in BW (≤ 150/5,000) and SL (≤ 200/2,000) or lose the concession to operate a gastronomic facility in SL. Only the smoke-free laws of HE, RP and SN explicitly enumerate a noncommercial private function as an exemption to the smoking ban in pubs and restaurants. In BY, the state constitutional court ruled that the statutory smoking ban does not apply to true private functions. In NW, the smoke-free law does not apply to premises used privately. The government of SL informs that the smoke-free law does not apply to true private functions hosted in a pub or restaurant because, from a legal point of view, no gastronomic facility is operating for the limited time of the private function. *Source:* Own compilation based on state smoke-free laws [12,13,14,15,16,17,18,19,20,21,22,23,24,25,26,27,28], 2005 and 2009 microcensus data [38], and supplementary information [9,31,32,33,34,37].

### 3.2. State Non-smoker Protection

Starting in 2007, each of the 16 federal states in Germany introduced a state law for the protection of non-smokers. By 1 July 2008, smoke-free laws were ratified in all federal states, but smoking became prohibited to a different extent in public places across states (Table 1). Smoking in pubs and restaurants, for instance, is banned throughout Germany. In most states, however, there are exemptions from the ban that allow smoking in separate rooms and in so-called *smoking pubs* that operate in less than 75 m^2^ and only admit people over 18 years old. Additional restrictions on the type of food that can be severed in smoking pubs may also apply. Strict no-smoking policies without exemption during public events in pubs and restaurants exist only in Bavaria, Saarland, and since 1 May 2013 also in North Rhine-Westphalia.

On 30 July 2008, the Federal Constitutional Court announced the leading decision that some of the just introduced smoking bans and their exemptions constitute a violation of the fundamental right to freely exercise a profession: Firstly, small gastronomic facilities, e.g., *corner pubs* (Eckkneipen), that focus on selling drinks and operate in one room, in which smoking is banned, are put at an economic disadvantage through the smoke-free law if larger gastronomic facilities are allowed to permit smoking in a separate room. Secondly, nightclubs are unduly disadvantaged if the smoke-free law allows gastronomic facilities but not nightclubs to establish separate smoking rooms. New undiscriminating regulations were to be issued by 31 December 2009 (BVerfG, 1 BvR 3262/07, 402/08, 906/08). This ruling occurred after two innkeepers and a nightclub operator had filed constitutional complaints against the non-smokers protection laws of Berlin and Baden-Wuerttemberg, respectively. The Federal Constitutional Court declared the concerned clauses of the smoke-free laws in these two states incompatible with the constitution and mandated to either eliminate or extend smoking ban exemptions in a manner that does not discriminate nightclubs or small gastronomic facilities. The objected smoke-free laws remained valid until revised within the transition period, but small gastronomic facilities in Baden-Wuerttemberg and Berlin could immediately readmit smoking if no food was prepared and sold on the premises, size was below 75 m^2^, no separate side-room existed, and minors were not admitted. In addition, a sign in the entrance area had to indicate that smoking is allowed and entry of minors is prohibited. Nightclubs in Baden-Wuerttemberg could immediately allow smoking in separate rooms without a dance floor if access was for adults only.

Formally, the ruling applied only to Berlin and Baden-Wuerttemberg, but other states were indirectly affected because their smoke-free laws included exemptions similar to the clauses of the two smoke-free laws ruled unconstitutional. In the opinion of the court, at this particular time, only the states of Bavaria and Saarland did regulate non-smokers protection conform to the German constitution. The Bavarian smoke-free law imposed stricter bans without exemptions for publicly accessible pubs and restaurants, and it did not list separate regulations for nightclubs. The smoke-free law of Saarland allowed the smoking ban exemptions of separate rooms and of *owner-operated gastronomic facilities* (inhabergeführte Gaststätte), and it allowed also nightclubs to permit smoking in separate rooms without a dance floor.

After this leading decision of the Federal Constitutional Court on two state smoke-free laws, most German states readmitted smoking under certain conditions in small gastronomic facilities that operated in one room only. The House of Representatives of Berlin, for instance, adopted the *First Amendment to its Non-smokers Protection Act* on 14 May 2009 [43]. So-called *smoking pubs* (Rauchergastätten), in which smoking was allowed, could be created in consequence. Primarily in Bavaria, in which the initial smoke-free law had completely ruled out smoking in all pubs another option was pursued. *Smoking clubs* (Raucherclubs) that required membership were formed because the initial smoke-free law of Bavaria did not ban smoking when a pub hosted a *private function* (geschlossene Gesellschaft). An amendment to the Bavarian smoke-free law from 27 July 2009, which was effective until 31 July 2010, eliminated the *smoking club* exemption and admitted *smoking pubs* and smoking in separate rooms [44]. A constitutional complaint against the smoke-free law of Bavaria was not accepted by the Federal Constitutional Court on 10 September 2009 after this amendment (BVerfG, 1 BvR 2054/09). On 31 January 2012, the Bavarian Constitutional Court ruled that the inclusion of *smoking clubs* in the smoking ban for pubs and restaurants was in line with the constitution [14], but that smoking can be admitted in *true private functions* (echte geschlossene Gesellschaften) (VerfGH Bayern, Vf. 26-VII-10). A true private function was characterized by the Bavarian Administrative Court as a gathering that is limited from the outset to a usually small number of personally invited individuals who are already known before the event, e.g., a private family celebration (VGH Bayern, 24 January 2011, 10 CS 11.2).

A further successful constitutional complaint against statutory smoking bans affected the state smoke-free law of Hamburg on 24 January 2012 (BVerfG, 1 BvL 21/11). The complaint objected to a discrimination among gastronomic facilities in Hamburg, where pubs (that focus on selling drinks) under certain conditions could permit smoking, but restaurants (that focus on selling food) could not. The state of Hamburg revised its non-smokers protection law accordingly and eliminated the condition that smoking could be allowed in separate rooms only if no food was prepared in the gastronomic facility.

**Figure 1:**
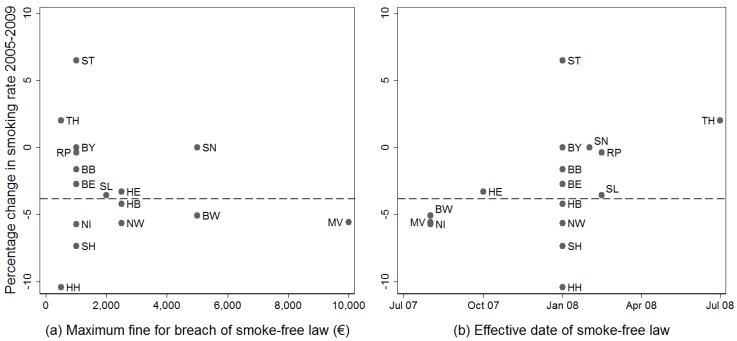
Maximum fine for a breach of the smoke-free law, effective date of the smoke-free law and change in smoking rate in German states.

Notes: The dashed line indicates the national average change in the smoking rate from 2005 to 2009 as a percentage of the baseline year 2005. (a) Spearman’s rank correlation coefficient ρ = −0.14 (*p* = 0.61). (b) Spearman’s rank correlation coefficient ρ = 0.51 (*p* = 0.04). Source: Own computation based on Table 1.

Although the focus of the public debate lies on smoking bans for pubs and restaurants (including cafes and snack bars), the smoke-free laws of the German states regulate smoking in further public places including nightclubs, cultural venues, schools, youth and daycare centers, hospitals, universities, sport centers, shopping malls, or state airports, courts and prisons. Notwithstanding how comprehensively a state banned smoking, neither the maximum fine that can be imposed for violations of a smoking ban nor the maximum fine that can be imposed for not enforcing the state smoke-free law correlates with a higher reduction in smoking (Spearman’s ρ = −0.01, *p* = 0.99 and Spearman’s ρ = −0.14, *p* = 0.61; Figure 1a). By contrast, an early effective date of smoking bans correlates with a higher percentage decrease in the smoking prevalence from 2005 to 2009 (Spearman’s ρ = 0.51, *p* = 0.04; Figure 1b). The latter correlation could be explained, for instance, by a delayed effect of later smoking bans on smoking behavior. It could also be accounted for by alternative explanations. We discuss two plausible alternative causes and arguments against them. Firstly, it is possible that those states with a higher reduction in smoking were more successful in implementing smoke-free laws early because of advantageous initial conditions affecting both. The past smoking rate of the state could be such an initial condition, but the 2005 smoking rates (implicitly reported in Table 1) appear to correlate neither with the effective dates of the first smoke-free laws (Spearman’s ρ = −0.15, *p* = 0.58) nor with the percentage change in the smoking rate from 2005 to 2009 across states (Spearman’s ρ = −0.31, *p* = 0.24). Secondly, the correlation between the change in the smoking rate and the ratification date of the first state smoke-free law could be due to generally more comprehensive tobacco control in the states adopting smoking bans early. However, we are not aware of other significant differences between states with regards to tobacco control activities apart from the state smoke-free legislation in Germany. An in depth investigation of the causal relationship underlying the observation that an early effective date of smoking bans is associated with a higher reduction in smoking in Germany is outside the scope of this article.

### 3.3. Petitions, Popular Initiatives and Referendums on Non-smokers Protection

In addition to the top-down legislation and the legal action of individuals, numerous initiatives by citizens’ or interest groups attempted to influence non-smokers protection legislation through the direct democratic procedures available in Germany. All of Germany’s state constitutions offer a referendum and some also a petition as instruments by which the citizens of the state can directly influence the democratic decision-making process. There are differences between states, but most states follow a three-stage process for the referendum. The referendum is preceded by a popular initiative, which needs to be preceded by a motion. A state parliament may debate an issue that passed through a motion or hear its initiators. A popular initiative has a higher quorum than a motion. If the quorum of the popular initiative is passed, it results in a referendum. A petition is different from a motion for a popular initiative. It is nonbinding and solely leads to a discussion of the matter in the state parliament if successful [45].

Initiatives on the issue of smoking in public places have been formed by individuals or interest groups in order to directly influence the smoke-free legislation (Table 2). The objectives of the various initiatives in favor of non-smokers protection in Germany were similar. They asked for a smoke-free environment that includes restaurants, bars and pubs, cafes, discos and clubs, playgrounds, and other shared spaces. Counterinitiatives rejected comprehensive smoking bans that provide few or no exemptions. So far, there have been 15 direct democracy initiatives leading to two petitions, 13 motions for a popular initiative, two popular initiatives and one referendum on non-smokers protection in Germany, all of which included the regulation of smoking in pubs and restaurants. Four initiatives were in favor of more comprehensive non-smokers protection in public places, and 11 initiatives against it. Seven of the 11 initiatives rejecting comprehensive smoking bans were initiated by one and the same initiators, *The Doers Society* (Die Macher e. V.), a registered association offering assistance with direct democratic procedures.

**Table 2:** Petitions, popular initiatives and referenda on non-smokers protection in Germany.

Federal State	Title or Aim of Direct Democracy Initiative	Initiators	Stage	Start–End	Signatories (Quorum)	Formal Success (Factual Success)
Bavaria	For real non-smokers protection (Für echten Nichtraucherschutz)	Action alliance of political parties, doctors, smoke-free society and others (Aktionsbündnis: ödp, Pro Rauchfrei e. V., Ärzte, SPD, Bündnis 90/Die Grünen, andere)	3	04.07.10	—	61% Approval rate (New smoke-free law [14])
			2	19.11.09–02.12.09	1,300,000 (940,000)	Yes
			1	01. 05.09–17.07.09	40,300 (25,000)	Yes ^a^
	Against the non-smokers protection law (Gegen das Nichtraucherschutzgesetz)	The doers society (Die Macher e.V)	1	26.01.08–January 2010	7,450 (25,000)	Ceased
Brandenburg	Free smokers (Freie Raucher)		1	16.01.08–November 2008	100 (20,000)	Ceased
Berlin	Fresh air for Berlin (Frische Luft für Berlin)	Smoke-free societies and others (Forum Rauchfrei, Nichtraucherbund Berlin-Brandenburg e. V., Pro Rauchfrei e. V., andere)	1	24.09.10–14.04.11	23,633 (20,000)	Yes (No) ^b,c^
	No smoking ban in pubs and restaurants in Berlin (Wahlfreiheit für Gäste und Wirte—kein Rauchverbot in Berliner Gaststätten)	Action alliance “Initiative for Consumption”—innkeepers (Aktionsbündnis “Initiative für Genuss”—Kneipen und Gastwirte)	2	26.01.09–25.05.09	61,644 (171,000)	No
			1	11.11.07–30.04.08	23,252 (20,000)	Yes
Hamburg	For true non-smokers protection—without exemptions (Für echten Nichtraucherschutz—ohne Ausnahmen)	Alliance of political party and smoke-free society (ödp, Nichtraucherschutz Hamburg e. V.)	1	05.07.10–05.01.11	<10,000 (10,000)	No
	Against the non-smokers protection law (Gegen das Nichtraucherschutzgesetz)	Initiative “Smoking Rebels Hamburg”—individual innkeepers (Initiative “Hamburger Rauchrebellen”—einzelne Gastwirte)	1	07.09.07–08.12.07	11,000 (10,000)	Yes (No) ^d^
Hesse	Against the revision of smoking bans (Gegen die Neuregelungen zum Rauchverbot)	The doers society (Die Macher e. V.)	1	10.12.07–February 2010	>50,000 (130,000)	Ceased ^a^
Lower Saxony	Reform non-smokers protection law—for exemptions (Reform Nichtraucherschutzgesetz—für Ausnahmeregelungen)	Hotel and restaurant association lower saxony (Hotel-und Gaststättenverband Niedersachsen)	1	26.11.07–25.11.08	66,210 (70,000)	No ^b^
North Rhine-Westphalia	For better non-smokers protection (Für verbesserten Nichtraucherschutz)	Action alliance of pharmacists and others (Aktionsbündnis: Apotheken und andere)	1	29.01.07–21.12.07	Not available	Ceased ^e^
	Against the non-smokers protection law (Gegen das Nichtraucherschutzgesetz)	The doers society (Die Macher e.V)	1	25.01.08–15.06.09	30 (3,000)	Ceased ^f^
Rhineland-Palatinate	Legalize smoking (Legalisierung von Rauchen)		1	07.02.08–31.12.08	5,500 (20,000)	Ceased
Saarland			1	07.02.08–15.06.09	50 (5,000)	Ceased
Schleswig-Holstein			1	01.01.08–15.06.09	8,150 (20,000)	Ceased
Thuringia			1	01.01.08–15.06.09	800(5,000)	Ceased

Notes: Dates are denoted in the format date month year. Dashed lines separate different stages of one initiative. Solid lines separate different initiatives. Numbers denote the stage of the initiative: 1 motion for a popular initiative (Volksinitiative bzw. Antrag auf Volksbegehren) or petition if indicated by superscript; 2 popular initiative (Volksbegehren); 3 referendum (Volksentscheid). Superscripts denote remarks: ^a^ signatories by end of the year; ^b^ nonbinding petition that can solely lead to a discussion of the matter in the state parliament (Volkspetition); ^c^ state parliament rejected the petition; ^d^ referendum not requested; ^e^ state parliament ratified a non-smokers protection act on 19 December 2007; ^f^ Federal Constitutional Court requested a revision of smoking bans on 30 July 2010. No direct democracy initiatives on non-smokers protection took place in BW, HB, MV, NI or SN since 2000. *Source:* Own compilation based on state websites and referendum reports 2000–2012 [35,36].

Two waves of initiatives can be distinguished. Twelve initiatives started in 2007 through early 2008, around the time when the first state smoking bans took effect in Germany. All of those but one opposed smoking bans. One motion against the introduction of smoking bans in pubs and restaurants in Berlin was successful. It lead to the next level of a popular initiative, but it then failed the quorum. All other initiatives failed because the motion or petition was either ceased, stopped, rejected, unsuccessful, or resolved by the ratification of a non-smokers protection law. The very first referendum for better protection of non-smokers in Germany was attempted in the state of North Rhine Westphalia. It was the initiative ceased when the state parliament approved a non-smokers protection act on 19 December 2007.

A second wave of three initiatives took place between 2009 and 2010, all of which aimed at strengthening the protection from secondhand smoke through extending smoking bans. The second wave started with a successful direct democratic change of the Bavarian smoke-free law. After 40,300 signatures had been collected in a motion in 2009, a referendum led to a plebiscite held on 4 July 2010 (Bavarian State Government, B II 2—G 58/09). The Bavarian citizens voted in favor of the suggested stronger non-smoking bill, entitled *Real Non-smokers Protection* (Für echten Nichtraucherschutz), with a majority of 61 percent and a voter turnout of 37.7 percent. In consequence, a new smoke-free law took effect on 1 August 2010 [14]. Alike the initial Bavarian smoke-free law, it includes a strict ban of smoking in all pubs and restaurants [13]. Thus, it eliminated the later introduced exemptions of *smoking pubs* and smoking in side rooms [44]. It further eliminated a smoking ban exemption for beer and wine pavilions, like the beer tents of the Oktoberfest, and continued to prohibit smoking during pseudo private functions hosted in pubs and restaurants. A constitutional complaint against this strict ban on smoking in Bavaria was rejected by the Federal Constitutional Court on 2 August 2010 (BVerfG, 1 BvR 1746/10).

The success of the direct democracy initiative in Bavaria inspired two other direct democracy initiatives for non-smokers protection that started in Berlin and Hamburg in 2010. In contrast to the Bavarian plebiscite, the petition *Fresh Air for Berlin* (Frische Luft für Berlin), which had the support of 23,633 signatories, was dismissed in the Berlin Senate on 20 June 2011. The majority of the senate did not see the need to strengthen the existing non-smokers protection in Berlin [46]. In Hamburg, the initiative *For True Non-smokers Protection without Exemptions* (Für echten Nichtraucherschutz—ohne Ausnahmen) did not reach the quorum.

The pronounced direct democratic activity on smoking bans in Germany reflects the controversy about smoking bans. An interesting question in this context is whether fragmented or partial smoking bans are particularly disliked? A tentative answer to this question is suggested by two studies that examined whether the comprehensiveness of smoke-free policies affects the support for smoking bans. Firstly, a correlation analysis of self-reported attitudes toward smoking bans in 27 countries of the European Union found that citizens of countries with a higher *Tobacco Control Scale* (TCS) score in 2007 showed higher support toward smoking bans in pubs (Spearman’s ρ = 0.50, *p* < 0.01), restaurants (ρ = 0.47, *p* = 0.01), offices and other indoor workplaces (ρ = 0.39, *p* = 0.12). The correlation between the TCS score for smoke-free public places and the support for smoking bans in pubs (Spearman’s ρ = 0.66, *p* < 0.00), restaurants (ρ = 0.61, *p* = 0.001), offices and other indoor workplaces (ρ = 0.46, *p* = 0.02) may be even stronger [47]. Secondly, a longitudinal multi-country study that focused on the support of smoke-free policies among smokers concluded that smoke-free policies have the potential to improve support once the policy is in place, and that this effect seems to be most pronounced with comprehensive smoking bans [48]. In 2007 and 2010, Germany scored 37 out of a total of 100 TCS points for its implementation of tobacco control policies, and it scored 2 and 11 out of a total of 22 TCS points for smoke-free public places, respectively [7,8]. Hence, the limited extent of smoking bans itself may be one of the causes of people’s demands for revisions of the smoke-free legislation in Germany. Other factors that have been associated with attitudes toward any type of smoking ban in Germany were smoking status, health risk ascribed to tobacco use and tobacco smoke, and self-rated health [49].

## 4. Conclusions

Non-smokers protection in the Federal Republic of Germany is governed by the federal *Law for Protection from the Hazards of Passive Smoking*, which includes the *Federal Non-smokers Protection Act* and amendments to previously existing laws. The explicit decision if and where a smoking ban is imposed is up to each state parliament for public places other than federal institutions, public transport or public train stations. Smoking bans can, and in Bavaria were, directly influenced by the citizens through direct democratic means.

The federal government structure and direct democratic participation in non-smokers protection legislation produced a diversity of local smoking bans and exemptions in Germany. Several studies investigated the effect of smoking bans in Germany [50,51,52,53,54,55], but the impact of the latest amendments by which some federal states of Germany created stricter smoke-free laws have yet to be assessed.

## Supplementary Files

Supplementary File 1 Supplementary Information (PDF, 130 KB)
