# Supplementary material for: Smoke-Free Laws and Direct Democracy Initiatives on Smoking Bans in Germany: A Systematic Review and Quantitative Assessment
Source: Int J Environ Res Public Health. 2014 Jan 3;11(1):685–700. doi: 10.3390/ijerph110100685 (PMC3924468; doi:10.3390/ijerph110100685)
Supplement: Supplementary File 1 — Supplementary Information (PDF, 130 KB) [file ijerph-11-00685-s001.pdf]

Supplementary Information

Smoke-Free Laws and Direct Democracy Initiatives on Smoking Bans in Germany: A Systematic Review and Quantitative Assessment

---

Table S1. German state smoke-free laws and amendments.

***State*/Law or Amendment**	**Date**	**Page**	**Name of Law or Amendment (in German)**
Baden-Wuerttemberg			
Smoke-free law [1]	25.07.07	337	Landesnichtraucherschutzgesetz
1. Amendment act	03.03.09	81	Gesetz zur Änderung des Landesnichtraucherschutzgesetzes
Bavaria			
Smoke-free law [2]	20.12.07	919	Gesetz zum Schutz der Gesundheit
1. Amendment act	22.07.08	465	Gesetz zur Änderung des Gesundheitsschutzgesetzes
2. Amendment act [3]	27.07.09	384	Gesetz zur Änderung des Gesundheitsschutzgesetzes
3. Smoke-free law [4]	23.07.10	314	Gesetz zum Schutz der Gesundheit
Berlin			
Smoke-free law [5]	16.11.07	578	Gesetz zum Schutz vor den Gefahren des Passivrauchens in der Öffentlichkeit
1. Amendment act	16.11.07	578	§ 8 Abs. 3 Nichtraucherschutzgesetz
2. Amendment act [6]	14.05.09	250	Erstes Gesetz zur Änderung des Nichtraucherschutzgesetzes
3. Amendment act	03.06.10	285	§ 34 Abs. 1 Wohnteilhabegesetz
Brandenburg			
Smoke-free law [7]	18.12.07	346	Gesetz zum Schutz vor den Gefahren des Passivrauchens in der Öffentlichkeit
1. Amendment act	27.05.09	156	Erstes Gesetz zur Änderung des Brandenburgischen Nichtrauchendenschutzgesetzes
2. Amendment act	15.07.10	1	Art. 2 Abs. 3 Gesetz zur Errichtung und Auflösung von Landesoberbehörden sowie zur Änderung von Rechtsvorschriften
Bremen			
Smoke-free law [8]	18.12.07	515	Bremisches Nichtraucherschutzgesetz
1. Amendment act	16.12.08	413	Gesetz zur Änderung des Bremischen Nichtraucherschutzgesetzes
2. Amendment act	24.01.12	24	Nr. 2.1 i.V.m. Anl. 1 (no. 2.1 in conjunction with annex 1) Bekanntmachung über die Änderung von Zuständigkeiten
3. Amendment act	11.27.12	505, 545	Zweites Gesetz zur Änderung des Bremischen Nichtraucherschutzgesetzes
4. Amendment act	06.25.13	297	Gesetz zur Änderung des Bremischen Nichtraucherschutzgesetzes

Table S1. *Cont.*

***State*/Law or Amendment**	**Date**	**Page**	**Name of Law or Amendment (in German)**
Hamburg			
Smoke-free law [9]	11.07.07	211	Hamburgisches Gesetz zum Schutz vor den Gefahren des Passivrauchens in der Öffentlichkeit
1. Amendment act	15.12.09	494	Art. 4 Gesetz zur Umsetzung der Föderalismusreform im Heimrecht
2. Amendment act	15.12.09	506	Gesetz zur Änderung des Hamburgischen Passivraucherschutzgesetzes
3. Amendment act	19.06.12	264	Zweites Gesetz zur Änderung des Hamburgischen Gesetzes zum Schutz vor den Gefahren des Passivrauchens in der Öffentlichkeit
Hesse			
Smoke-free law [10]	06.09.07	568	Gesetz zum Schutz vor den Gefahren des Passivrauchens
1. Amendment act	14.12.09	666	Art. 4 Abs. 7 Hessisches Hochschulgesetz und Gesetz zur Änderung des TUD-Gesetzessowie weiterer Rechtsvorschriften
2. Amendment act	04.03.10	86	Gesetz zur Änderung des Hessischen Nichtraucherschutzgesetzes
3. Amendment act	27.09.12	290	Art. 11 Siebtes Gesetz zur Verlängerung der Geltungsdauer und Änderung befristeter Rechtsvorschriften
Mecklenburg-Western Pomerania			
Smoke-free law [11]	12.07.07	239	Nichtraucherschutzgesetz Mecklenburg-Vorpommern
1. Amendment act	17.12.09	738	Erstes Gesetz zur Änderung des Nichtraucherschutzgesetzes Mecklenburg-Vorpommern
Lower Saxony			
Smoke-free law [12]	12.07.07	337	Niedersächsisches Nichtraucherschutzgesetz
1. Amendment act	10.12.08	380	Gesetz zur Änderung des Niedersächsischen Nichtraucherschutzgesetzes
North. Rhine-Westphalia			
Smoke-free law [13]	20.12.07	742	Gesetz zum Schutz von Nichtraucherinnen und Nichtrauchern in Nordrhein-Westfalen
1. Amendment act	30.06.09	390	Gesetz zur Änderung des Gesetzes zum Schutz von Nichtraucherinnen und Nichtrauchern in Nordrhein-Westfalen
2. Amendment act	04.12.12	635	Gesetz zur Änderung des Nichtraucherschutzgesetzes NRW
Rhineland-Palatinate			
Smoke-free law [14]	05.10.07	534	Nichtraucherschutzgesetz Rheinland-Pfalz
1. Amendment act	26.05.09	205	Landesgesetz zur Änderung des Nichtraucherschutzgesetzes Rheinland-Pfalz
Saarland			
Smoke-free law [15]	21.11.07	75	Gesetz zum Schutz vor den Gefahren des Passivrauchens
1. Court decision	01.12.08	131	Entscheidung des Verfassungsgerichtshofs des Saarlandes (VerfGH Saarland, Lv 2/08, Lv 3/08, Lv 6/08), published in 2009
2. Amendment act	14.01.09	396	Gesetz zur Änderung des Nichtraucherschutzgesetzes und des Ersten Gesetzes zur Ausführung des Kinder- und Jugendhilfegesetzes

Table S1. *Cont.*

State/**Law or Amendment**	**Date**	**Page**	**Name of Law or Amendment (in German)**
3. Amendment act	06.05.09	906	§ 22 Abs. 3 Saarländisches Gesetz zur Sicherung der Wohn-, Betreuungs- und Pflegequalität für ältere Menschen sowie pflegebedürftige und behinderte Volljährige
4. Amendment act	10.02.10	25	Gesetz zur Änderung des Nichtraucherschutzgesetzes
5. Court decision	21.06.10	1236	Entscheidung des Verfassungsgerichtshofs des Saarlandes (VerfGH Saarland, Lv 3/10, Lv 4/10, Lv 6/10)
Saxony			
Smoke-free law [16]	26.10.07	495	Gesetz zum Schutz von Nichtrauchern im Freistaat Sachsen
1. Amendment act	26.06.09	318	Art. 3 Gesetz zur Neufassung des Gesetzes über Spielbanken im Freistaat Sachsen und zur Änderung des Sächsischen Ausführungsgesetzes zum Glücksspielstaatsvertrag
2. Amendment act	10.12.09	682	Gesetz zur Änderung des Sächsischen Nichtraucherschutzgesetzes
3. Amendment act	03.07.11	198	Art. 2 Sächsisches Gesetz zur Neuordnung des Gaststättenrechts
4. Amendment act	14.06.12	270	Art. 6 Gesetz zum Ersten Glücksspieländerungsstaatsvertrag, zum Staatsvertrag über die Gründung der GKL Gemeinsame Klassenlotterie der Länder und zur Änderung des Sächsischen Ausführungsgesetzes zum Glücksspielstaatsvertrag sowie weiterer Gesetze
Saxony-Anhalt			
Smoke-free law [17]	19.12.07	464	Gesetz zur Wahrung des Nichtraucherschutzes im Land Sachsen-Anhalt
1. Court decision	22.10.08	396	Entscheidung des Landesverfassungsgerichts Sachsen-Anhalt (LVerfG Sachsen-Anhalt, LVG 3/08, LVG 4/08, LVG 7/08, LVG 8/08)
2. Amendment act	14.07.09	373	Gesetz zur Änderung des Nichtraucherschutzgesetzes
3. Amendment act	17.02.11	136	§ 37 Abs. 3 Gesetz über Wohnformen und Teilhabe des Landes Sachsen-Anhalt
4. Amendment act	23.01.13	38	Art. 4 Gesetz zur Änderung des Kinderförderungsgesetzes und anderer Gesetze
Schleswig-Holstein			
Smoke-free law [18]	10.12.07	485	Gesetz zum Schutz vor den Gefahren des Passivrauchens
1. Amendment act	25.04.09	222	Gesetz zur Änderung des Gesetzes zum Schutz vor den Gefahren des Passivrauchens
Thuringia			
Smoke-free law [19]	20.12.07	257	Thüringer Gesetz zum Schutz vor den Gefahren des Passivrauchens
1. Amendment act	26.06.10	250	Erstes Gesetz zur Änderung des Thüringer Nichtraucherschutzgesetzes
2. Amendment act	02.07.12	245	Zweites Gesetz zur Änderung des Thüringer Nichtraucherschutzgesetzes

Notes: Dates are denoted in the format date month year. Officially announced changes of the state smoke-free laws were considered at least until 28 June 2013 and at most until 27 November 2013. Page denotes the starting page of the law in the *Law and Ordinance Gazette* of the state. § denotes a paragraph, Art. denotes a section, Abs. denotes a subsection of the law. Source: Own compilation based on Beck-Online [20].

References

1. Landesnichtraucherschutzgesetz (State non-smokers protection act). *Gesetzesblatt für Baden-Württemberg.* **2007**, 337–339.
2. Gesetz zum Schutz der Gesundheit (Gesundheitsschutzgesetz) (Law to protect health (Health protection act)). *Bayerisches Gesetz- und Verordnungsblatt* **2010**, 314–316.
3. Gesetz zur Änderung des Gesundheitsschutzgesetzes (Law to change the health protection act). *Bayerisches Gesetz- und Verordnungsblatt* **2009**, 384.
4. Gesetz zum Schutz der Gesundheit (Gesundheitsschutzgesetz) (Law to protect health (Health protection act)). *Bayerisches Gesetz- und Verordnungsblatt* **2007**, 919–921.
5. Gesetz zum Schutz vor den Gefahren des Passivrauchens in der Öffentlichkeit (Nichtraucherschutzgesetz) (Law to protect against the dangers of passive smoking in public (Non-smokers protection act)). *Gesetz- und Verordnungsblatt für Berlin* **2007**, 578–579.
6. Erstes Gesetz zur Änderung des Nichtraucherschutzgesetzes (First act to amend the non-smokers protection law). *Gesetz- und Verordnungsblatt für Berlin* **2009**, *13*, 250.
7. Gesetz zum Schutz vor den Gefahren des Passivrauchens in der Öffentlichkeit (Brandenburgisches Nichtrauchendenschutzgesetz) (Law to protect against the dangers of passive smoking in public (Brandenburg non-smokers protection act)). *Gesetz- und Verordnungsblatt für das Land Brand.* **2007**, *I*, 346–347.
8. Bremisches Nichtraucherschutzgesetz (Bremen non-smokers protection act). *Gesetzblatt der Freien Hansestadt Bremen* **2007**, 515–517.
9. Hamburgisches Gesetz zum Schutz vor den Gefahren des Passivrauchens in der Öffentlichkeit (Hamburgisches Passivraucherschutzgesetz) (Hamburgian law to protect against the dangers of passive smoking in public (Hamburgian passive- smokers protection act)). *Hamburgisches Gesetz- und Verordnungsblatt* **2007**, *I*, 211–212.
10. Gesetz zum Schutz vor den Gefahren des Passivrauchens (Hessisches Nichtraucherschutzgesetz) (Law to protect against the dangers of passive smoking (Hessian non-smokers protection act)). *Gesetz- und Verordnungsblatt für das Land Hessen* **2007**, *I*, 568.
11. Nichtraucherschutzgesetz Mecklenburg-Vorpommern (Non-smokers protection act Mecklenburg-western Pomerania). *Gesetz- und Verordnungsblatt für Mecklenburg-Vorpommern* **2007**, 239–240.
12. Niedersächsisches Gesetz zum Schutz vor den Gefahren des Passivrauchens (Niedersächsisches Nichtraucherschutzgesetz) (Lower Saxony law to protect against the dangers of passive smoking (Lower Saxony non-smokers protection act)). *Niedersächsisches Gesetz- und Verordnungsblatt* **2007**, 337–338.
13. Gesetz zur Verbesserung des Nichtraucherschutzes in Nordrhein-Westfalen (Law to improve non-smokers protection in north Rhine-Westphalia). *Gesetz- und Verordnungsblatt für das Land Nordrhein-Westfalen* **2007**, 742–743.
14. Nichtraucherschutzgesetz Rheinland-Pfalz (Non-smokers protection act Rhineland-Palatinate). *Gesetz- und Verordnungsblatt für das Land Rheinland-Pfalz* **2007**, 534–536.

15. Gesetz zum Schutz vor den Gefahren des Passivrauchens (Nichtraucherschutzgesetz) (Law to protect against the dangers of passive smoking (Non-smokers protection act)). *Amtsblatt des Saarlandes* **2008**, *I*, 75–78.
16. Gesetz zum Schutz von Nichtrauchern im Freistaat Sachsen (Sächsisches Nichtraucherschutzgesetz) (Law on the protection of non-smokers in the free state of Saxony (Saxon non-smokers protection act)). *Sächsisches Gesetz- und Verordnungsblatt* **2007**, 495–496.
17. Gesetz zur Wahrung des Nichtraucherschutzes im Land Sachsen-Anhalt (Nichtraucherschutzgesetz) (Law to preserve non-smokers protection in the state of Saxony-Anhalt (Non-smokers protection act)). *Gesetz- und Verordnungsblatt für das Land Sachsen-Anhalt* **2007**, 464–465.
18. Gesetz zum Schutz vor den Gefahren des Passivrauchens (Law to protect against the dangers of passive smoking). *Gesetz- und Verordnungsblatt für Schleswig-Holstein* **2007**, 485–486.
19. Thüringer Gesetz zum Schutz vor den Gefahren des Passivrauchens (Thuringian law to protect against the dangers of passive smoking). *Gesetz- und Verordnungsblatt für den Freistaat Thüringen* **2007**, 257–258.
20. Verlag, C.H. Beck-online—Die Datenbank (The Database). Available online: http://beck-online.beck.de (accessed on 2 December 2013).

© 2014 by the authors; licensee MDPI, Basel, Switzerland. This article is an open access article distributed under the terms and conditions of the Creative Commons Attribution license (http://creativecommons.org/licenses/by/3.0/).
